# Haemodynamic Recovery Properties of the Torsioned Testicular Artery Lumen

**DOI:** 10.1038/s41598-017-15680-3

**Published:** 2017-11-14

**Authors:** Selda Goktas, Ozlem Yalcin, Erhan Ermek, Senol Piskin, Can T. Capraz, Yusuf O. Cakmak, Kerem Pekkan

**Affiliations:** 1 Koc University, Mechanical Engineering Department, Istanbul, 34450 Turkey; 2 Koc University, Department of Physiology, Istanbul, 34450 Turkey; 3 Otago University, Department of Anatomy, Dunedin, 9054 New Zealand

## Abstract

Testicular artery torsion (twisting) is one such severe vascular condition that leads spermatic cord injury. In this study, we investigate the recovery response of a torsioned ram testicular artery in an isolated organ-culture flow loop with clinically relevant twisting modes (90°, 180°, 270° and 360° angles). Quantitative optical coherence tomography technique was employed to track changes in the lumen diameter, wall thickness and the three-dimensional shape of the vessel in the physiological pressure range (10–50 mmHg). As a control, pressure-flow characteristics of the untwisted arteries were studied when subjected to augmented blood flow conditions with physiological flow rates up to 36 ml/min. Both twist and C-shaped buckling modes were observed. Acute increase in pressure levels opened the narrowed lumen of the twisted arteries noninvasively at all twist angles (at ∼22 mmHg and ∼35 mmHg for 360°-twisted vessels during static and dynamic flow experiments, respectively). The association between the twist-opening flow rate and the vessel diameter was greatly influenced by the initial twist angle. The biomechanical characteristics of the normal (untwisted) and torsioned testicular arteries supported the utilization of blood flow augmentation as an effective therapeutic approach to modulate the vessel lumen and recover organ reperfusion.

## Introduction

Torsion of the testicles due to twisting of the spermatic cord is a severe vascular condition that impacts one in 4000 males who are less than 25 years of age^1^. Twisting of the spermatic cord impedes the blood supply to the testicles, and in order to avoid ischaemic injury and infarction, the patient should be operated on immediately^2^. Prolonged diagnosis-to-surgery times may prove very risky in case of testicular artery (TA) (i.e., main spermatic cord artery) twisting, as it can lead to testicular loss especially in adolescents and neonates. Moreover, post-operative blood flow problems are also common after the detorsioning surgery^3^. Indeed, knowledge of the microvascular haemodynamics of TA is critical to understand the ischaemic torsional configurations, and to eliminate the possibility of post-operative reperfusion injuries^4^.

The degree of the spermatic cord twist and duration of the torsion determine the magnitude of testicular damage. The extent of torsion can vary from 90° to three complete turns of the spermatic cord^5^. Recently, extensive investigatory efforts have aimed to find effective strategies to minimize, or possibly prevent, testicular injury following testicular torsion. As an example to those attempts, Acar *et al*. demonstrated that peripheral nerve stimulation could improve testicular perfusion in a 180° testicular torsion model of rats^6^. In another study, electro-stimulation at 10 Hz frequency has been shown to have a significant effect on increasing the volume flow rate and diameter of the human internal artery, whereas electro-stimulation at 80 Hz had opposite effects^7^. Thus, response of the blood flow rate to external electro-stimulation depends on the patient characteristics, stimulation region, stimulation duration and frequency.

Torsional buckling is a common configuration that is generated due to the relative twist of vessel ends^8^. Likewise, a topologically C-shaped buckling is also likely when the internal pressure of vessel segments exceeds a mechanical limit^9^. Additionally, the C-shaped buckling mode may occur through torsional deformation due to the narrowed vessel cross sectional area^10^. Unfortunately, for a twisted TA, it is still unclear which mode of buckling is more prevalent clinically, since *in vivo* experimental studies focusing on the haemodynamic effects with live vessels of millimetre size are limited in the literature^11,12^, despite the existence of several perfusion studies on uniform undeformed large artery systems^13,14^.

Therefore, in this study, we primarily aim to characterize the haemodynamic interactions of the TA at different twist angles. The secondary aim is to assess the limitations of recently reported intervention techniques to alleviate and recover the twisted TA lumen. We hypothesize that the three-dimensional (3D) vessel lumen response of live TAs may be quantitatively predicted from *in vitro* haemodynamic flow models. The ram model was adopted for our experiments due to the close resemblance of its TA diameter to humans^15^. To the best of our knowledge, this study is the first to document a detailed analysis of TA buckling covering the multiple physiological twist modes *in vitro*.

## Results

### Healthy undeformed vessel characteristics

**Figure 1:**
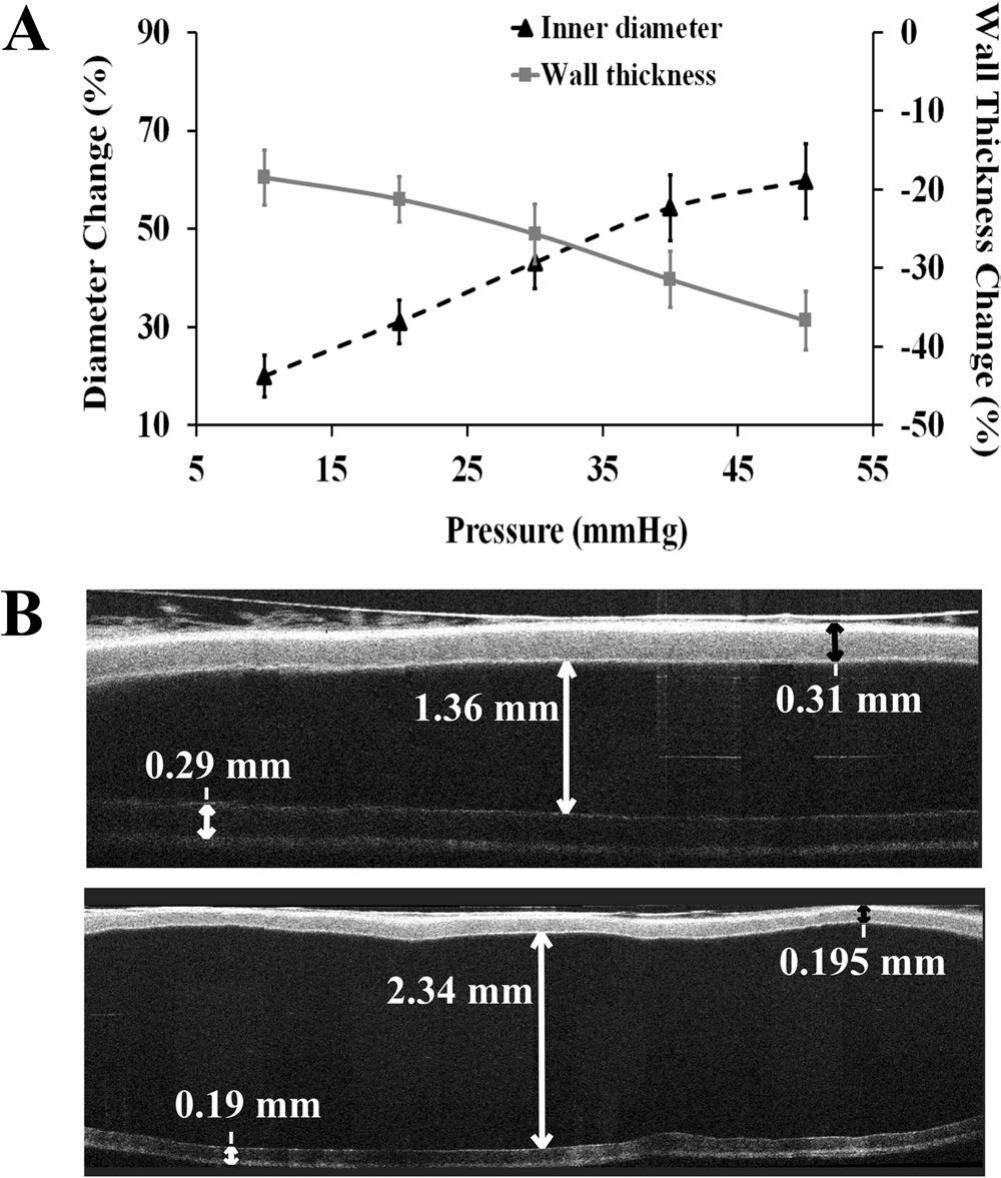
Dimensional changes in the untwisted TAs following intramural pressure increase. (**A**) The ID and WT values were obtained using the OCT system. Error bars correspond to the variations in vessel samples (*n* = 7) with individual vessel outer diameters as 2.26 mm, 1.82 mm, 2.07 mm, 1.94 mm, 2.07 mm, 1.49 mm, and 2.24 mm. All tested vessels were between 19–20 mm in length. (**B**, above) shows OCT image for the vessel with no pressure applied (0 mmHg) under static conditions. (**B**, below) illustrates the same vessel with 40 mmHg static axial pressure applied. The ID and WT values are indicated with red arrows.

As a first step, the compliance response of a straight untwisted live TA was tested using the optical coherence tomography (OCT) system. As the intramural pressure increased gradually, the inner diameter (ID) change for the TA segments (*n* = 7, average outer diameter = 1.98 ± 0.27 mm) was more than 50% through 10–50 mmHg (Fig. 1A). ID increased linearly up to 40 mmHg pressure and then displayed the characteristic nonlinear behaviour after this threshold value. As expected, the vessel wall thickness (WT) decreased with increasing pressure. Total decrease in WT was more than 35% at 50 mmHg pressure. The vessel ID increased from 1.36 mm under no applied pressure to 2.34 mm with decreasing WT throughout the vessel under 40 mmHg pressure, as seen from the OCT images (Fig. 1B).

**Figure 2:**
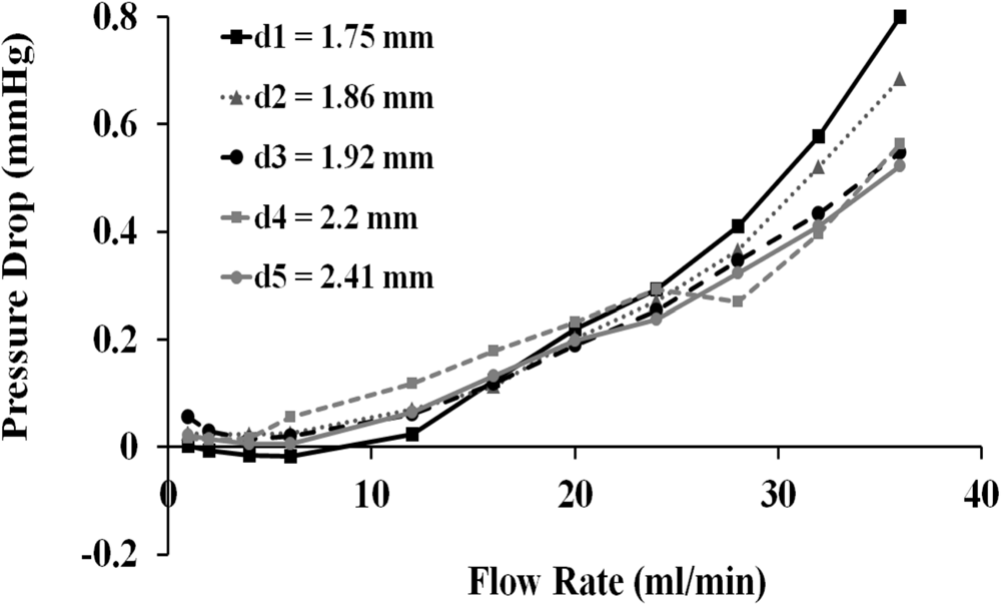
Pressure drop values measured from the dynamic flow experiments of an untwisted TA. Only the pressure drop due to the arterial segment was plotted, contributions from the auxiliary hydraulic components were excluded. Vessel outer diameters and lengths are as follows: d_1_ = 1.75 mm (L_1_ = 18.4 mm), d_2_ = 1.86 mm (L_2_ = 19.1 mm), d_3_ = 1.92 mm (L_3_ = 19.3 mm), d_4_ = 2.2 mm (L_4_ = 18.7 mm), and d_5_ = 2.41 mm (L_5_ = 18.5 mm).

Dynamic flow experiments were used to analyse the pressure drop variation with respect to flow rate for vessels (*n* = 5) without any twist (Fig. 2). In general, the predicted pressure drop values increased with increasing flow rate. Notably, pressure drop values were found to be higher in larger vessels for flow rates below 15 ml/min. This trend reversed when the flow rates were increased further up to 36 ml/min, which aligned with our theoretical computations (please see Supplementary Information).

### Torsional buckling and its release

**Figure 3:**
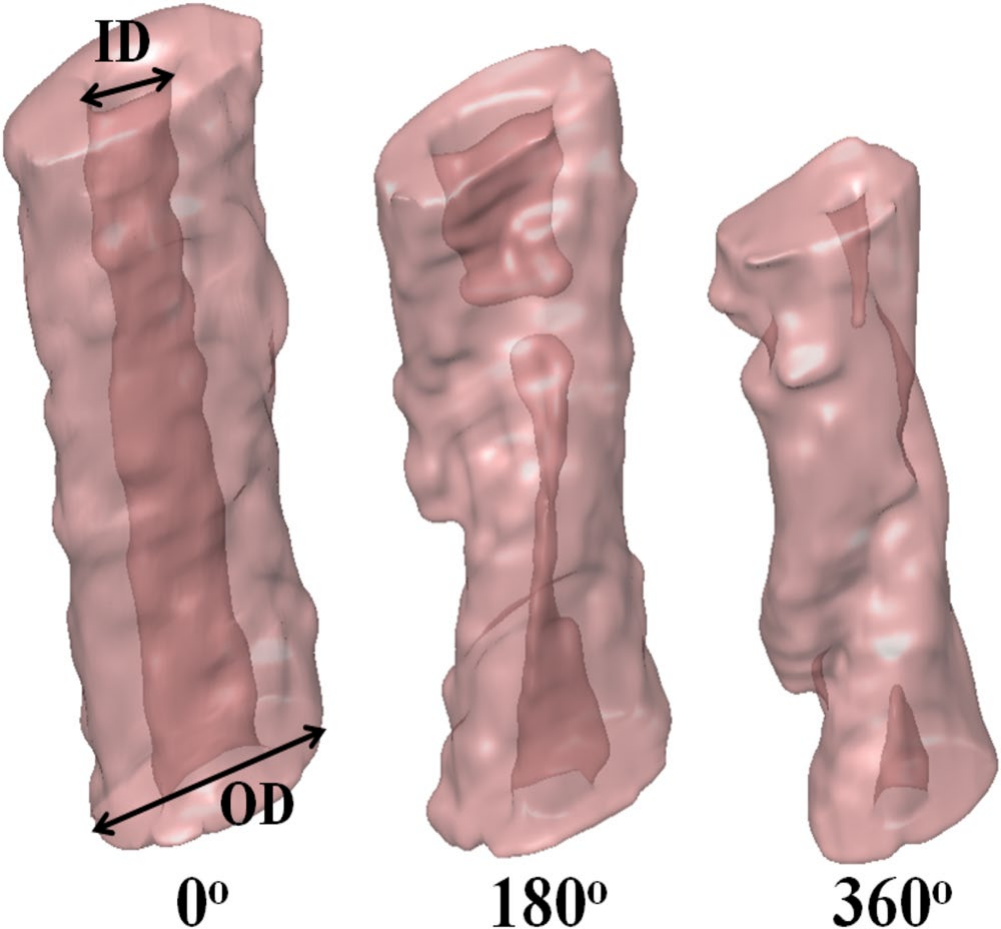
Reconstructed image data for the buckled TAs was obtained through OCT. ID and outer diameter (OD) of the vessels are labelled. The buckling obstructed intramural space and subsequent length reduction can be observed for 180° and 360°.

In Fig. 3, 3D reconstructions of twisted vessels are provided for the three torsional buckling modes of 0°, 180°, and 360°. For the 0° configuration, the intramural space is fully open, while for the 360° buckling configuration, the lumen is almost totally blocked. The 180° buckling mode corresponds to the initiation of total flow blockage and narrowing of the vessel lumen. Furthermore, a gradual decrease in vessel diameter and axial shortening are seen for higher buckling angles.

**Figure 4:**
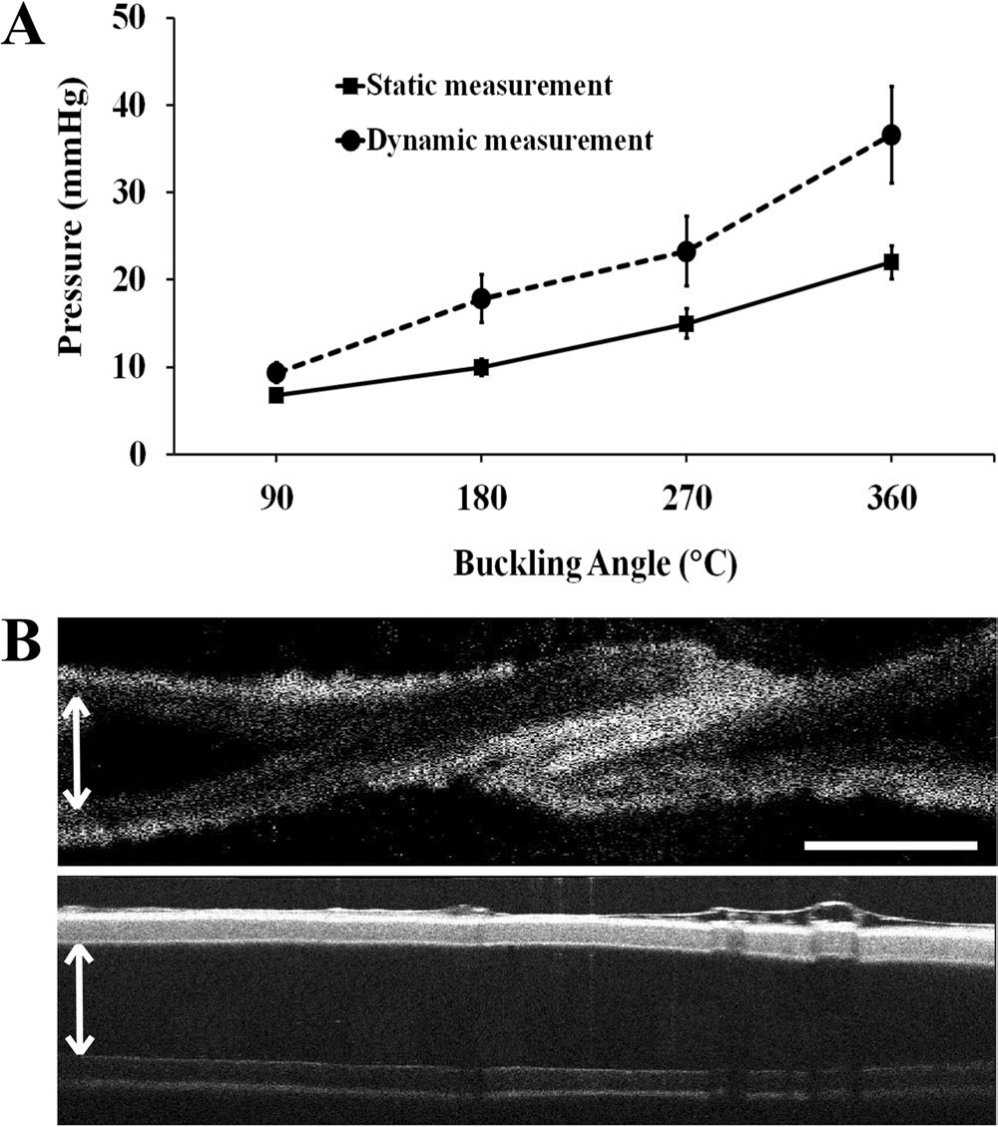
Opening of the twisted test samples with applied external pressure. (**A**) Pressure values needed for twist opening with respect to initial buckling angles for different vessels. Tested sample sizes were as follows: *n* = 5 at 90°, 180° and 270°, *n* = 4 at 360° for dynamic experiments; *n* = 6 at 180° and 360°, *n* = 4 at 90° and 270° for static test conditions. All tested vessels were between 19–20 mm in length. (**B**, above) illustrates OCT image of a vessel twisted with 270° under 0 mmHg pressure under static conditions, where first the vessel ID decreased, then a kink occurred. (**B**, below) shows that a pressure of 12 mmHg was required to completely remove the kink in the corresponding vessel while increasing the lumen diameter, shown by the white arrow. Scale bar = 2 mm.

The intramural pressure required to enlarge and open the stenosis caused by the torsional buckling is provided for increasing twist angles: 90°, 180°, 270° and 360° under both static and dynamic conditions (Fig. 4A). The general trend for both conditions was that for higher twist angles, higher pressures were needed to unbuckle the vessels. Noticeably, pressure readings at each buckling angle deviated more for dynamic measurements compared to static measurements. In addition, as the twist angles increased, more variations were observed in the opening pressure values between different vessels during both experiments. Figure 4B presents an illustrative OCT image as evidence to the unbuckling process with increased pressure during static experiments.

**Figure 5:**
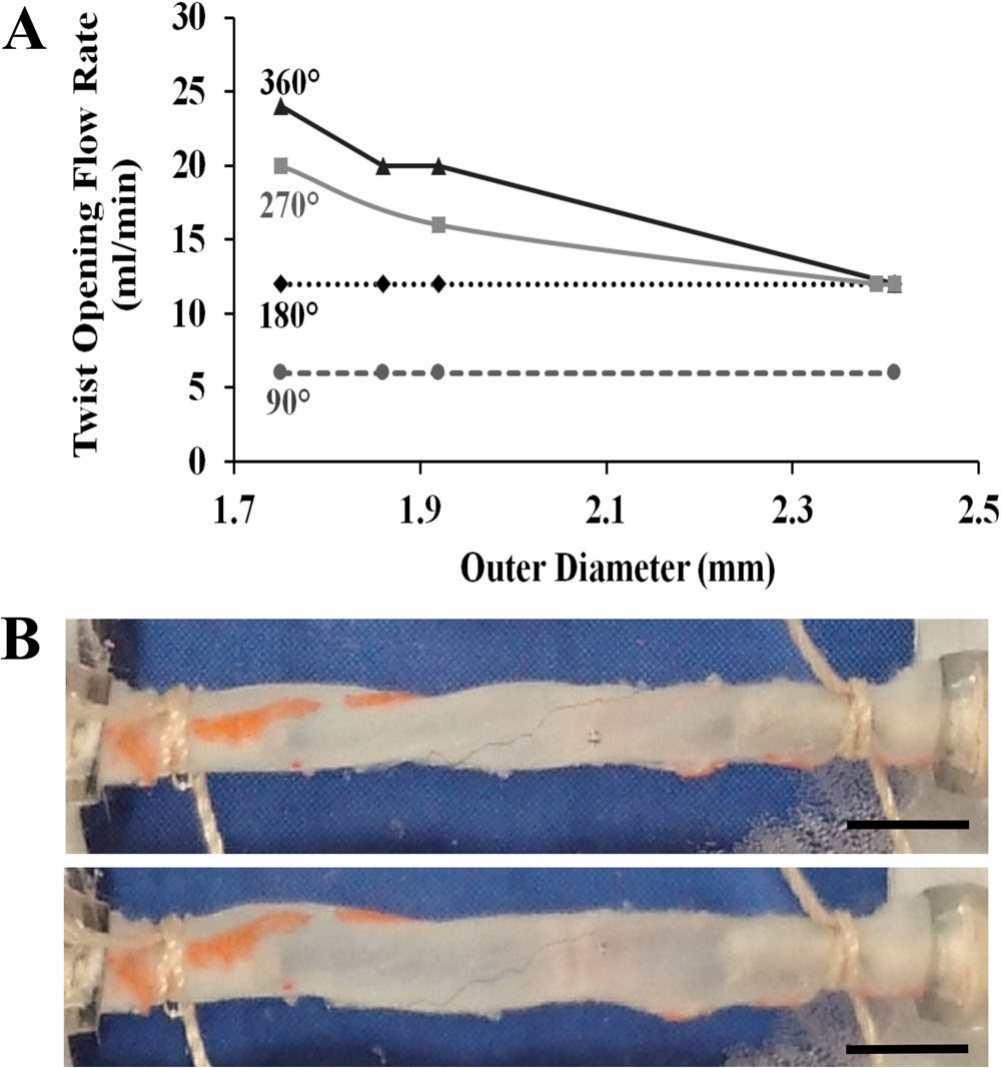
Opening of the twisted TAs in response to increased flow. (**A**) The changes of twist opening flow rate in response to varying outer vessel diameter for different twist angles (90°, 180°, 270° and 360°) are presented. Four different TAs were tested (*n* = 4). All tested vessels were between 19–20 mm in length. (**B**, above) presents a test vessel twisted at 270°. (**B**, below) shows the twist opening of this vessel with flow. The vessel twisting and opening were tracked with orange tissue marking dye. Scale bar designates 2 mm.

In dynamic flow conditions, high flow rates are required to sustain the intramural pressure needed to keep the twisted vessel open. Two different trends were observed depending on the initial twist angle (Fig. 5A). For small twist angles such as 90° and 180°, twist opening flow rate remained constant, irrespective of the vessel diameter. On the other hand, for higher twist angles (270° and 360°), the twist opening flow rate demonstrated a decreasing trend in response to increasing diameter. Interestingly, all twist angles except 90°, the twist opening flow rate values converged to the same flow rate level for a vessel diameter of 2.4 mm. The twisting of a vessel at 270°, and its subsequent opening with flow are illustrated in Fig. 5B.

### C-shaped buckling characteristics

**Figure 6:**
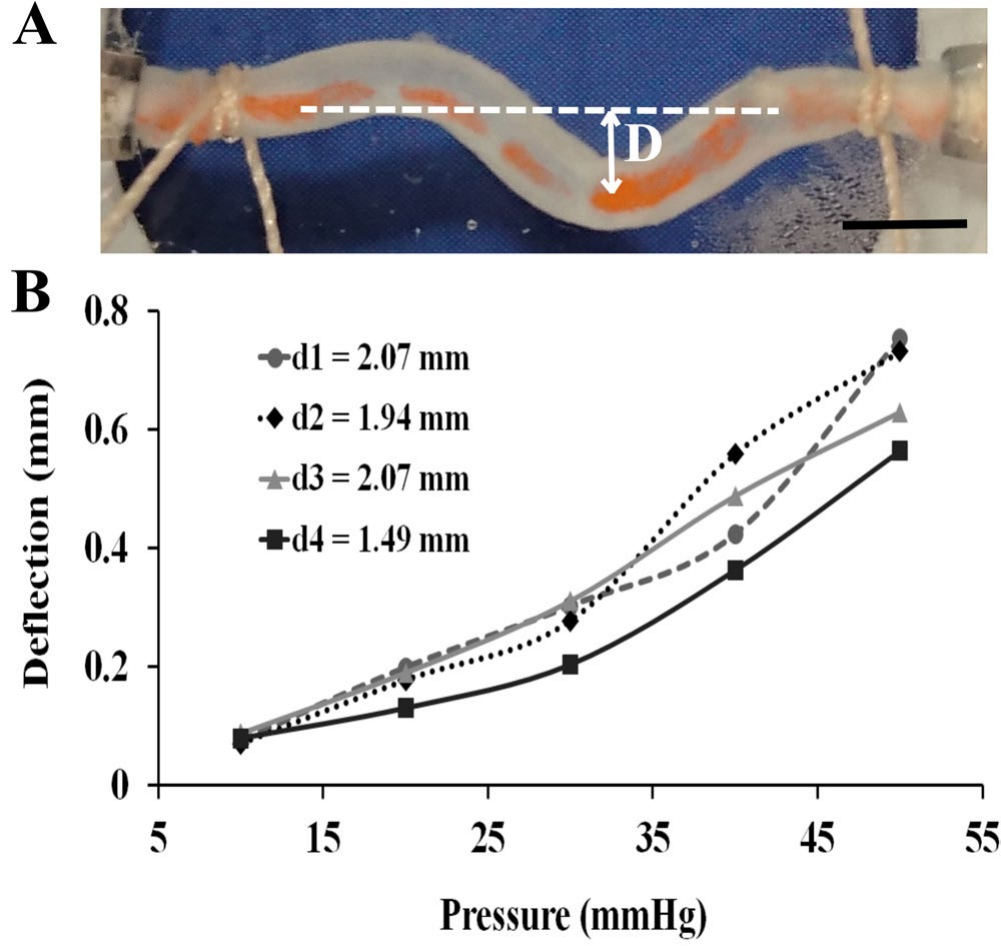
Deflection in the artery segments with increasing intramural pressures. (**A**) Deflection (**D**) of the artery from the centreline (depicted with the white dashed line) due to C-shaped buckling is shown with the white arrow (scale bar, 2 mm). (**B**) The pressure-deflection trend is plotted for four TA segments with outside diameters and lengths as d_1_ = 2.07 mm (L_1_ = 19.5 mm), d_2_ = 1.94 mm (L_2_ = 19.4 mm), d_3_ = 2.07 mm (L_3_ = 18.3 mm), and d_4_ = 1.49 mm (L_4_ = 19 mm).

C-shaped buckling mode was detected for untwisted TAs when the intramural pressure levels within the vessels were further increased above critical statically (Fig. 6A). Similar conditions were observed for increased flow rates during dynamic experiments. Deflection from the centreline for the C-shaped buckled vessels increased with increasing pressure (*n* = 4, Fig. 6B). At lower pressures, the vessels’ deflection behaviour closely resembled each other. However, above 30 mmHg pressure, the varying response of the arteries in terms of deflection was noticeable.

**Figure 7:**
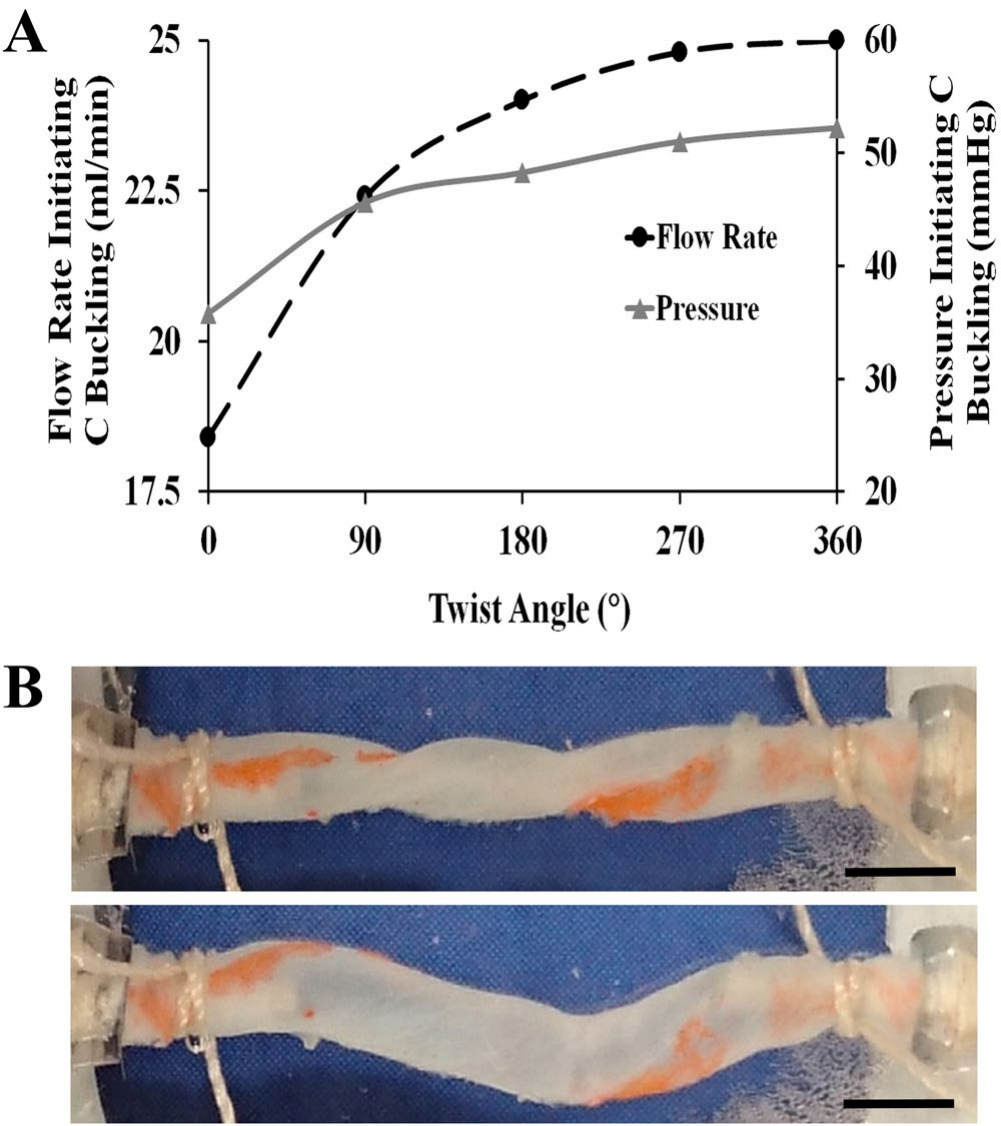
C-buckling mode following the opening of the vessels twisted at various degrees. (**A**) The average flow rate and pressure values required to initiate C-buckling within the TAs. No twist condition (i.e., 0° twisting) was used as control. Tested sample sizes were as follows: *n* = 5 at 0°, 90°, 180° and 270°, *n* = 4 at 360°. All tested vessels were between 19–20 mm in length. (**B**, above) shows a TA first twisted at 360°. (**B**, below) indicates the C buckling mode for the corresponding artery after opening the twist. Scale bar represents 2 mm.

Figure 7A presents the haemodynamic conditions that initiate C-shaped buckling for 5 artery samples that were first twisted at various angles. Under applied pressure and flow rate, the buckling opens-up on its own, without any other intervention. Further increases in flow rate and pressure cause the C-shaped buckling state. Both the flow rate and the pressure required to start the C buckling mode within the arteries depicted an increasing trend with increasing twist angle. The flow rate values started to plateau at a twisting angle of 360 °C. An illustrative image for the initiation of C-buckling is illustrated in Fig. 7B, where a vessel was first twisted at 360°; following the opening of the vessel, as flow rate increased, C-shaped buckling was observed.

## Discussion

In large arteries, experimental and theoretical studies have shown that twist buckling starts when lumen pressure exceeds the critical pressure at a given length^16^. Other possible biomechanical factors in the aetiology of arterial buckling are high blood pressure (hypertension), reduced wall elasticity due to lamina/elastic fibre degradation, decreased critical pressure and axial stretch/tension^17–19^. However, detailed haemodynamic characteristics of buckled millimetre-sized arteries, such as TAs, have not been studied in the literature.

Modulation of intramural pressure and flow can be employed to remove the torsional buckling acutely. Our results documented the flow rate necessary to open the buckled TA increased as the twist angle increased. We have also presented the quantitative association between the opening pressures of the twisted arteries and the twist angles (Fig. 4). As the twist angle increased, the opening pressure of the artery lumen had an increasing trend for all test specimens. That might be because higher torque is generated on the artery at high twist rates, as shown in the study by Garcia *et al*.^20^. In their study, they torsioned porcine common carotid arteries at four different axial stretch ratios (SR), and reported increased buckling torques for increased SR. We estimated that this ratio was very low in our case, and therefore assumed a constant value (see Limitation section for further detail). It should be emphasized that, in an intact testicle, the tissue mass around the buckled arterial segment would require an additional flow volume/pressure which will exceed the predictions of the present *in vitro* study. While this contribution is believed to be low due to small distal mass, detailed mechanical tests are needed for its exact value.

Removal of buckling in the TA can also be achieved through an increase in the arterial diameter. The study by Cakmak *et al*. showed the ID of the human TA increased from 1.65 mm to 2.03 mm in 5 minutes under 10 Hz frequency external electro-stimulation^15^. The same study reported the volumetric flow rate augmentation for the corresponding diameter changes as 8 ml/min and 17 ml/min, respectively. In our study, Fig. 5A shows that a flow rate of 17 ml/min is indeed sufficient to open 90°, 180° and 270° buckling of the TAs with an approximately equivalent diameter of 2 mm. Although the two studies were performed on different model systems, and the diameter values were measured differently, the flow rate and pressure scales of humans and rams were found to be very close to each other. However, due to significant differences in the length of the test sample (L_sample_) versus the actual human artery length (L_human_), the corresponding torsional angles (θ) should be adjusted through a linear relation for small angles; θ_human_ = θ_sample_.L_human_/L_sample_. When we extrapolated human data^15^ to our volumetric flow data, diameter and the degree of torsion (Fig. 5A), we could conclude that the increase in volume flow may increase pressure, and thus may open the ~180° buckled arteries in humans. We can also suggest that the same technique could be effective for torsions up to 360°, but not for torsions any higher than that. Unfortunately, in the current clinical setting, urologists have no diagnostic preoperative imaging techniques to determine what degree of torsion a testicle has, and it is anecdotally believed that torsional levels reaching 720° is not uncommon. For this reason, when used in clinics, the proposed mechanism to open a buckled artery should involve a trial-and-error protocol of gradually increasing blood flow. Hence, more studies need to be conducted to validate the consistency of the stimulation technique and related volume flow and diameter changes.

To obtain the relationship between flow rate and pressure drop, the pressure drop measurements were processed with our theoretical model, which calculates head losses in the dynamic setup (see Supplementary Information for details). This model extracted the pressure drop values in the TA for corresponding flow rates and vessel diameters. Pressure drops increased in response to increased flow rates, as expected from the Poiseuille flow calculations. Our pressure drop results were consistent with the human TA findings in the literature. We calculated a pressure drop of 0.21 mmHg in 1 cm of artery under 18.84 ml/min flow rate (Fig. 2), while Waites and Moule found the corresponding pressure drop as 0.208 mmHg^21^.

Khalafvand and Han have shown on the porcine carotid artery that the critical buckling pressure does not change with the flow rate under steady state conditions^22^. In addition, the pulsatile flow decreased this critical buckling value by 17–23%. In line with their findings, our experiments demonstrated that pressure was the more influential haemodynamic parameter. The influence of wall shear stress created by blood flow on the arterial geometry was found to be minor. Another result from the same study was that a pressure change of 50 mmHg caused the middle of the carotid artery to deflect 30 mm, whereas we observed approximately 0.8 mm of deflection in our test samples for the same pressure change. Considering that our TA specimen was ∼5 times shorter than their carotid artery, we may conclude that increasing the specimen length would also increase the degree of deflection. Our results confirmed a substantial increase in TA deflection following the initiation of C-shaped buckling within the pressure range of 10 and 50 mmHg.

The luminal pressure increase should be controlled for the vessels that are likely to undergo C-shaped buckling after opening the torsional buckling. In particular, arteries with smaller twist angles have lesser critical C-shaped buckling pressures due to the lesser torque load on the artery. In our study, C-shaped buckling was observed in all vessels of various diameters with different initial twist angles after a critical flow rate of >20 mL/min was reached. This level of flow rate should be avoided to eliminate C-shaped buckling after detorsion of the twisted vessel. The resistance in peripheral capillaries could also be decreased to prevent this buckling mode. However, such substantial decrease in capillary resistance would also decrease the total pressure in the vessel lumen, and may propagate torsional buckling due to low pressure levels. Our results for critical buckling angle in relation with the vessel lumen pressure data could help determine the optimal pressure levels as a preliminary guide for further studies on the therapeutic methodologies targeting the recovery of twisted arteries.

### Limitation and Conclusion

The arteries, *in vivo*, operate under a finite SR that decreases by aging and as a result of vascular diseases. This ratio has a substantial effect on the critical buckling pressure and surface deflection. For instance, an increase in SR from 1.3 to 1.5 results in a difference in critical buckling pressure of up to 10–15 mmHg^22^. Since we avoided stretching the vessels in the axial direction, we employed very low SRs as 1.1 and 1.3 in our experiments. An increase in vessel diameter due to a low axial SR under lumen pressure would reduce the twist angle needed to reach the shear strain for buckling to occur. Our experiments were performed for outer diameters varying between 0.175 cm and 0.24 cm. That is a relatively narrow range for human TA. For comparative purposes, a TA with larger diameter could be tested in future studies.

In dynamic experiments, a theoretical model was used to extract the pressure drop in the isolated TA segment. For each hydrodynamic component of the set-up, the corresponding head loss formulas are highly sensitive to small vessel diameters and taper angle variations between the connections. Since our vessels were not fully cylindrical, and the connections were not arranged in a perfectly symmetric way, it was not possible to measure each connection parameter accurately. Furthermore, the pressure loss was calculated under the assumption of Poiseuille model, although the flow in the dynamic set-up was not fully developed due to the short inlet region and curved (deformed) pipe and artery geometry. Despite this assumption, our results are within the physiological range, and are thus significant for clinical estimates.

The current study elucidates the pressure-flow interactions and the corresponding dynamic surface deformation patterns of the torsioned TAs under different twist angles in a controlled organ culture system. The proposed haemodynamic approach offers a non-invasive alternative to the standard scrotal exploration and orchidopexy treatments but its clinical adoption requires parallel developments in imaging and optimization of clinical protocol alternatives. These findings, based on live TA, suggest a novel therapeutic approach in which the augmentation of volume flow can increase lumen pressure, resulting in opening and rescue of the twisted arteries in a twist angle dependent manner.

## Methods

### Sample preparation

Fresh TAs were collected from 32 healthy male white rams (4–6 months old) at a local slaughterhouse, and placed in cold-oxygenated physiological salt solution (PSS) of the following composition: mM; NaCl 119, KCl 4.7, NaHCO_3_ 24, KH_2_PO_4_ 1.18, MgSO_4_.7H_2_O 1.17, CaCl_2_ 1.6 and glucose 5.5. Arterial segments were gently isolated from surrounding fat and connective tissue to outer diameters of 1.70 to 2.45 mm under a dissecting microscope.

### Experimental protocol

**Figure 8:**
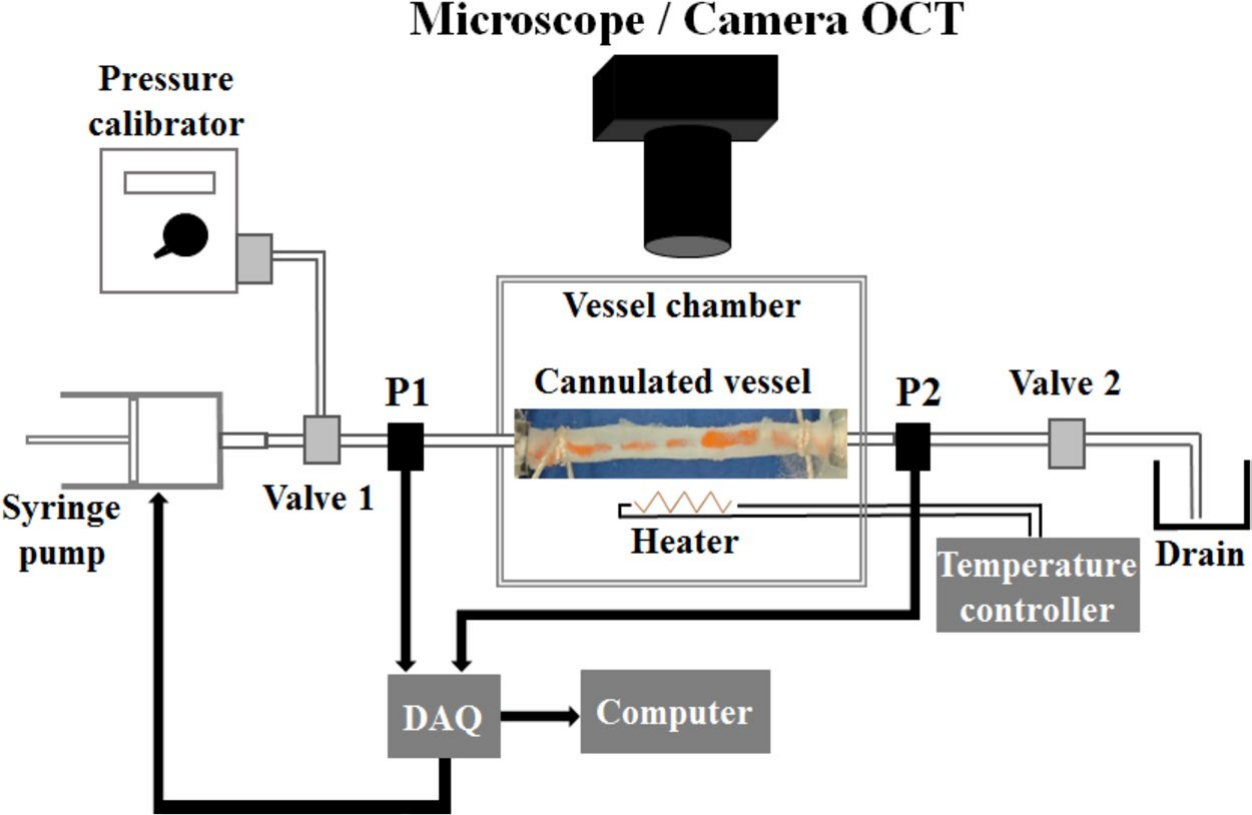
*In vitro* artery organ culture with schematic components of the static and dynamic experiment systems. The pressure within the vessels was adjusted by varying flow inside the vessels using inlet and outlet valves, Valve 1 and Valve 2, respectively. P1 and P2, pressure transducers; DAQ, data acquisition.

The arterial segment preparations (1.8–2 cm long) were transferred to a physiological vessel chamber (CH/1, Living Systems; Burlington, VT) that is filled with oxygenated (95% O_2_/5% CO_2_) PSS, and cannulated between two glass micropipettes with nylon ties (Fig. 8). The pH in the bath was maintained at 7.4 by adjusting the reservoir gassing rate. A heating element was included in the vessel chamber to maintain the set temperature of 37 °C. The chamber was placed under an inverted microscope equipped with a camera that was connected to LabVIEW software (NI Labview 2015) and a high-speed data acquisition card (National Instrument Systems DAQ 6221-BNC) to control the perfusion rate of the syringe pump, and to read the inlet (P1) and outlet (P2) pressure data.

The set-up was employed for both static (i.e. no flow) and dynamic flow experiments. For preconditioning, the arterial segments were allowed to equilibrate at 30 mmHg intraluminal pressure and zero flow rate for 30 min before the start of both experiments. At the end of the equilibration period, the presence of functional vessel segments was confirmed by checking the ability of acetylcholine (10^−6^ M) to produce relaxation of vessels precontracted with phenylephrine (10^−7^–10^−5^ M)^23^.

In the static experiments, a closed pressure system was maintained by filling the inside of the TA with PSS and closing the outflow stopcock valve (Valve 2, Fig. 8). Pressure was applied up to 50 mmHg. Arterial WT and inner (lumen) diameter of the vessels were acquired at each increment of 10 mmHg pressure increase through an OCT imaging system. Measurements were conducted both for the undeformed, and for the 90°, 180°, 270° and 360° twisted artery segments. When the twisted arteries were opened and reached the untwisted, initial (starting) diameter under pressure loading, the corresponding pressure values were recorded. Any further pressure increase beyond these “twist opening pressure” values was monitored for possible C-shaped buckling within the artery.

In the dynamic experiments, eleven different flow rate values, from 1 ml/min to 36 ml/min, were tested. Automated readout measurements of outer vessel diameter (within 5% error with the LabVIEW software), inlet/outlet pressure data (P1 and P2, respectively) and flow rate control of the syringe pump were recorded every 100 ms (Fig. 8). 60 pressure data points were collected for each flow rate, of which 40 were deemed reliable. Averages of the P1 and P2 pressure data were taken for corresponding flow rates and used to estimate twist opening pressures and pressure drop values. Due to the finite distance from the vessel sample, the physiological pressure drop values at the vessel sample were obtained by incorporating the theoretical pressure drop values of auxiliary set-up elements using Poiseuille flow assumption, orifice equations and sudden expansion formulas. In addition, flow rates of C-shaped buckling and twist opening were noted. This protocol was repeated for the above-mentioned twist angles. The twist angles were manually adjusted, and with the aid of a tissue marking dye, twist offset direction and angles were tracked. Moreover, the 3D lumen shapes of the twisted TA samples were reconstructed following our previously published protocols^24^.

### Data availability

All data generated or analysed during this study are included in this published article.

## Electronic supplementary material

Supplementary Information
