# Supplementary material for: Haemodynamic Recovery Properties of the Torsioned Testicular Artery Lumen
Source: Sci Rep. 2017 Nov 14;7:15570. doi: 10.1038/s41598-017-15680-3 (PMC5686114; doi:10.1038/s41598-017-15680-3)
Supplement: Supplementary file 1 — Supplementary Information [file 41598_2017_15680_MOESM1_ESM.pdf]

SUPPLEMENTARY INFORMATION

Haemodynamic Recovery Properties of the Torsioned Testicular Artery Lumen

Selda Goktas[1], Ozlem Yalcin[2], Erhan Ermek[1], Senol Piskin[1], Can T. Capraz[1], Yusuf O. Cakmak[3,+], Kerem Pekkan[1,+,*]

[1] Koc University, Mechanical Engineering Department, Istanbul, 34450, Turkey

[2] Koc University, Department of Physiology, Istanbul, 34450, Turkey

[3] Otago University, Department of Anatomy, Dunedin, 9054, New Zealand

[*] kpekkan@ku.edu.tr

[+] these authors contributed equally to this work

Calculations of pressure drop and flow rate

Under the assumption of Poiseuille flow, blood flow velocity, flow rate and the corresponding pressure drop values in testicular artery (TA) were calculated as in equation (1):

$$\Delta P = \frac{8 \cdot \mu \cdot L \cdot Q}{\pi \cdot r^4} \tag{1}$$

where ΔP is the pressure drop (mmHg), μ the dynamic viscosity (mmHg.s), L the length of the artery (mm), Q the volumetric blood flow rate (mm^3/s) and r is the radius (mm) of the TA. For a given pressure drop value, the Q value can easily be extracted from the above equation.

The average velocity (v_{ave}) (mm/s) within the vessel thus can be computed using equation (2) as follows:

$$Q = v_{ave} \,. A \tag{2}$$

with A as the vessel cross-sectional area (mm^2). Since the TA is long enough, we can use the assumption of developed flow within the artery. Thus, the velocity profile is paraboloid and the maximum velocity ($v_{\max}$) can be calculated as in equation (3):

$$v_{max} = 1.5 \cdot v_{ave} \tag{3}$$

Besides verifying our results, equations (1) and (2) allowed us to calculate the change in volume flow rate and pressure drop with respect to the narrowing of the artery in case of buckling. Our assumption was validated by the simulation results, where the pressure drop decreased as the vessel diameter increased for a constant flow rate of 0.188 ml/min, and the flow rate increased for a constant average velocity of 0.1 m/s, as presented in Supplementary Fig. S1.

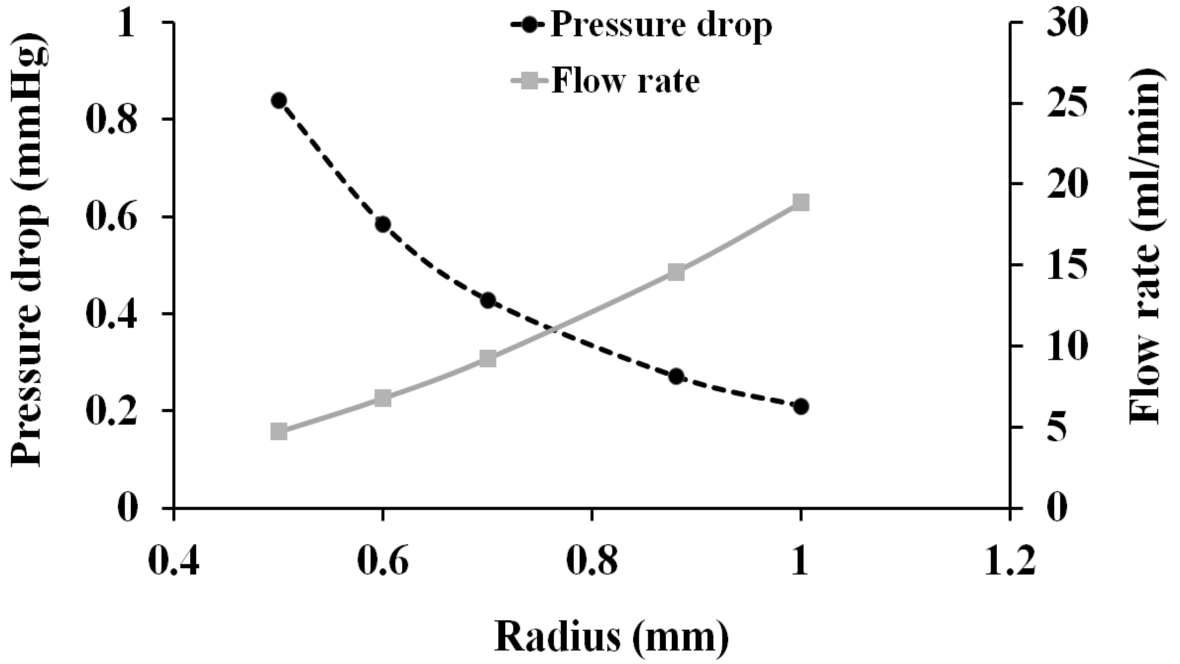

Supplementary Figure S1 | Estimated pressure drop-volume flow rate relation in an idealized, untwisted TA. Poiseuille flow assumption was applied to calculate the pressure drop and flow rate values for the tested vessels. The length and radius values were obtained from our experimental measurements.

After validating our model, we then performed computer simulations in our dynamic experimental set-up. Pressure drops measured in dynamic experiments are extremely higher than that of physiological range due to the design and limitations of the measurement system. The system includes many auxiliary elements besides the testicular artery: the connector arms to hold the artery, the connector pipes, C-shaped extension pipes and transducer connection apparatuses. Connections between these elements have sudden or tapered expansions and reductions which cause high pressure drops compared to that caused by the TA itself. Therefore, we have designed a theoretical (analytical) model to predict the pressure drops caused by each auxiliary element

and to find out the pressure drop within the TA. The contribution of the elements to the pressure drop values was estimated using standard head loss calculations under the assumption of Poiseuille flow for all vessel specimens and flow rates used in the measurements. The MATLAB code used to simulate the pressure drop values that are presented in Fig. 2 is as follows:

```
%%
% This code predicts the pressure drop throughout a test device
% with several auxiliary equipment such as pipe area expansion or
% reduction connections. All predictions are based on laminar flow
% assumption through circular pipe.

% Each part of this code calculates the head loss due to a connection
% within the system. The variables are explained next to their
% initializations in the corresponding section. All components are assumed to
% be rigid

%%
clear all;clc;

% Global variables which are valid for all equipment are defined in this
% part
rho=1060; mu=8.9e-4; %density and viscosity of the blood
g=9.81; % gravitational acceleration
Qvarray=[1 2 4 6 12 16 20 24 28 32 36]; % Flow rate array
Dvarray=[1.32 1.91 1.15 1.6 1.26]*1E-3; % Diameter array
thetaarray=[35 55 3 43 44]; % Connection angles of auxiliary test equipment

for i=1:11
%%
Qv=Qvarray(i); Qv=Qv*1e-6/60; %volumetric flow rate mL/min and m^3/s
Q=Qv*rho; %mass flow rate
Dv=Dvarray(5); %vessel diameter (m)

%%
% Calculation of pressure drop for a sudden pipe size reduction from radius
% r1 to r2 with an average velocity of V.

% head loss calculation for square reduction
r1=Dv/2;r2=0.6E-3/2; a1=pi*r1^2; a2=pi*r2^2;
V=(Qv/a1+Qv/a1)/2; %average velocity
r=(r1+r1)/2;
Re=rho*V*2*r/mu; %Reynolds number
K=(1.2+160/Re)*( (r1/r2)^4-1 );
hl1=K*V^2/(2*g);
hl1=hl1*9804.139432;

%%
% Calculation of pressure drop for a smooth pipe size reduction from
% radius r1 to r2 with an average velocity of V. Here the cross sectional
% area reduction is not sudden but with an inclination defined by theta
```

```
% angle.

%if the reduction is smooth (tapered)
theta=35; %taper angle for all connections
theta2=thetaarray(5); %taper angle for vessel only
hl1=1.6*sin(theta2/2*pi/180)*hl1;

%%
% Calculation of pressure drop for a sudden pipe size increase from radius
% r1 to r2 with an average velocity of V. All pipes and connection have
% circular shape.

% head loss calculation for square expansion
r1=0.6E-3/2;r2=Dv/2; a1=pi*r1^2; a2=pi*r2^2;
K=2*(1-(r1/r2)^4); V=(Qv/a1+Qv/a2)/2;
hl1_2=K*V^2/(2*g);
hl1_2=hl1_2*9804.139432;

%%
% Calculation of pressure drop for a smooth pipe size increase from
% radius r1 to r2 with an average velocity of V. Here the cross sectional
% area increase is not sudden but with an inclination defined by theta
% angle.

%if the expansion is smooth (tapered)
theta2=thetaarray(5); %taper angle for vessel only
hl1_2=2.6*sin(theta2/2*pi/180)*hl1_2;

%%
% For a straight circular pipe with laminar flow, the pressure drop is
% calculated in this part with r1 representing the pipe radius and
% L the pipe length

% pressure drop along the pipe
V=(Qv/a1+Qv/a1)/2;
Re=rho*V*2*r1/mu; %reynolds number
fD=64/Re; %friction factor
L=14e-3; %length of pipe
dp1=L*fD*rho*V^2/(2*2*r1);
% 1 meter of head loss is equal to 9804.139432

%%
% Calculation of pressure drop for a sudden pipe size increase from radius
% r1 to r2 with an average velocity of V. All pipes and connection have
% circular shape.

% head loss calculation for square expansion
r1=0.5E-3/2;r2=1.1E-3/2; a1=pi*r1^2; a2=pi*r2^2;
K=2*(1-(r1/r2)^4); V=(Qv/a1+Qv/a2)/2;
hl2=K*V^2/(2*g);
hl2=hl2*9804.139432;

%%
% Calculation of pressure drop for a smooth pipe size increase from
% radius r1 to r2 with an average velocity of V. Here the cross sectional
```

```
% area increase is not sudden but with an inclination defined by theta
% angle.

%if the expansion is smooth (tapered)
theta2=32;
hl2=2.6*sin(theta/2*pi/180)*hl2;

%%
% Calculation of pressure drop for a sudden pipe size reduction from radius
% r1 to r2 with an average velocity of V.

% head loss calculation for square reduction
r1=1.1E-3/2;r2=0.5E-3/2; a1=pi*r1^2; a2=pi*r2^2;
K=(1.2+160/Re)*( (r1/r2)^4-1 ); V=(Qv/a1+Qv/a1)/2;
hl2_2=K*V^2/(2*g);
hl2_2=hl2_2*9804.139432;

%%
% Calculation of pressure drop for a smooth pipe size reduction from
% radius r1 to r2 with an average velocity of V. Here the cross sectional
% area reduction is not sudden but with an inclination defined by theta
% angle.

%if the reduction is smooth (tapered)
theta2=32;
hl2_2=1.6*sin(theta/2*pi/180)*hl2_2;
r2=1.1E-3/2; a2=pi*r2^2;

%%
% For a straight circular pipe with laminar flow, the pressure drop is
% calculated in this part with r1 representing the pipe radius and
% L the pipe length

% pressure drop along the pipe
V=Qv/a2; %average velocity
Re=rho*V*2*r2/mu; %reynolds number
fD=64/Re; %friction factor
L=45e-3; %length of pipe
dp2=L*fD*rho*V^2/(2*2*r2);

%%
% Calculation of pressure drop for a sudden pipe size reduction from radius
% r1 to r2 with an average velocity of V.

% head loss calculation for square reduction
r1=1.1E-3/2;r2=1.0E-3/2; a1=pi*r1^2; a2=pi*r2^2;
K=(1.2+160/Re)*( (r1/r2)^4-1 ); V=(Qv/a1+Qv/a1)/2;
hl3=K*V^2/(2*g);
hl3=hl3*9804.139432;
%if the reduction is smooth (tapered)
theta2=32;
hl3=1.6*sin(theta/2*pi/180)*hl3;

%%
% Calculation of pressure drop for a sudden pipe size increase from radius
```

```
% r1 to r2 with an average velocity of V. All pipes and connection have
% circular shape.

% head loss calculation for square expansion
r1=1.0E-3/2;r2=1.1E-3/2; a1=pi*r1^2; a2=pi*r2^2;
K=2*(1-(r1/r2)^4); V=(Qv/a1+Qv/a2)/2;
hl3_2=K*V^2/(2*g);
hl3_2=hl3_2*9804.139432;

%%
% Calculation of pressure drop for a smooth pipe size increase from
% radius r1 to r2 with an average velocity of V. Here the cross sectional
% area increase is not sudden but with an inclination defined by theta
% angle.

%if the expansion is smooth (tapered)
theta2=32;
hl3_2=2.6*sin(theta/2*pi/180)*hl3_2;
r2=1.0E-3/2; a2=pi*r2^2;

%%
% For a straight circular pipe with laminar flow, the pressure drop is
% calculated in this part with r1 representing the pipe radius and
% L the pipe length

% pressure drop along the pipe
V=Qv/a2; %average velocity
Re=rho*V*2*r2/mu; %Reynolds number
fD=64/Re; %friction factor
L=15e-3; %length of pipe
dp3=L*fD*rho*V^2/(2*2*r2);

%%
% Calculation of pressure drop for a sudden pipe size increase from radius
% r1 to r2 with an average velocity of V. All pipes and connection have
% circular shape.

% head loss calculation for square expansion
r1=1.0E-3/2;r2=3.0E-3/2; a1=pi*r1^2; a2=pi*r2^2;
K=2*(1-(r1/r2)^4); V=(Qv/a1+Qv/a2)/2;
hl4=K*V^2/(2*g);
hl4=hl4*9804.139432;

%%
% Calculation of pressure drop for a smooth pipe size increase from
% radius r1 to r2 with an average velocity of V. Here the cross sectional
% area increase is not sudden but with an inclination defined by theta
% angle.

%if the expansion is smooth (tapered)
theta2=32;
hl4=2.6*sin(theta/2*pi/180)*hl4;

%%
% Calculation of pressure drop for a sudden pipe size reduction from radius
```

```
% r1 to r2 with an average velocity of V.

% head loss calculation for square reduction
r1=3.0E-3/2;r2=1.0E-3/2; a1=pi*r1^2; a2=pi*r2^2;
K=(1.2+160/Re)*( (r1/r2)^4-1 ); V=(Qv/a1+Qv/a1)/2;
hl4_2=K*V^2/(2*g);
hl4_2=hl4_2*9804.139432;

%%
% Calculation of pressure drop for a smooth pipe size reduction from
% radius r1 to r2 with an average velocity of V. Here the cross sectional
% area reduction is not sudden but with an inclination defined by theta
% angle.

%if the reduction is smooth (tapered)
theta2=32;
hl4=1.6*sin(theta/2*pi/180)*hl4;

%%
% For a straight circular pipe with laminar flow, the pressure drop is
% calculated in this part with r1 representing the pipe radius and
% L the pipe length

% pressure drop along the pipe
r1=3.0E-3/2;r2=2.0E-3/2; a1=pi*r1^2; a2=pi*r2^2;
r=(r1+r2)/2;
V=(Qv/a1+Qv/a2)/2; %average velocity
Re=rho*V*2*r/mu; %Reynolds number
fD=64/Re; %friction factor
L=30e-3; %length of pipe
dp4=L*fD*rho*V^2/(2*2*r);

% Here, all the pressure drops due to pipe flow and cross-sectional area
% change are summed and converted into mmHg from Pa.

total1=hl1+dp1+hl2+dp2+hl3+dp3+hl4+dp4;
total2=hl1_2+dp1+hl2_2+dp2+hl3_2+dp3+hl4_2+dp4;
total(i)=total1+total2;
total(i)=total(i)/133.322365; %convert Pa to mmHg
end
total=total'
```
